# Antiglomerular basement membrane antibody type rapidly progressive glomerulonephritis with seizures: Two cases and literature review

**DOI:** 10.1002/iid3.1074

**Published:** 2023-11-08

**Authors:** Chongyang Han, Xiangrong Cui, Zhicheng Tan, Yafeng Li, Yufeng Qiao

**Affiliations:** ^1^ Department of Nephrology Shanxi Provincial People's Hospital (Fifth Hospital) of Shanxi Medical University Taiyuan China; ^2^ Shanxi Provincial Key Laboratory of Kidney Disease Taiyuan China; ^3^ Reproductive Medicine Center, The Affiliated Children's Hospital of Shanxi Medical University, Children's Hospital of Shanxi Shanxi Maternal and Child Health Hospital Taiyuan China

**Keywords:** antiglomerular basement membrane disease, epilepsy, reversible posterior leukoencephalopathy syndrome, seizure, uremic encephalopathy

## Abstract

**Background:**

Rapidly progressive glomerulonephritis (RPGN) is clinically manifestations as a rapidly progressive renal failure and pathologically as crescentic and necrotizing lesions with infiltration of inflammatory cells in the glomeruli. Uremic encephalopathy (UE) usually develops in patients who are suffering from acute or chronic renal failure.

**Objective:**

The purpose of this article is to provide reference for clinical diagnosis and treatment of renal disease complicated with seizures. Patients Two cases of anti‐glomerular basement membrane type rapidly progressive glomerulonephritis complicated with seizures were reported.

**Materials & Methods:**

In case 1, a 40‐year‐old woman was hospitalized for the treatment of nausea, anorexia, and fever. On admission, she presented with elevated serum inflammatory indicators, moderate anemia, and advanced acute kidney injury requiring hemodialysis. Her anti‐glomerular basement membrane (GBM) antibody in serum and renal tissues was found to be extremely high. She was finally diagnosed with anti‐GBM disease. She was treated with a combination of corticosteroid pulse therapy, oral cyclophosphamide and prednisolone, and plasma exchange, while continued to require maintenance hemodialysis for end‐stage kidney disease. During treatment, she suddenly suffered blindness, seizure, and consciousness disturbance. She was diagnosed as posterior reversible leukoencephalopathy syndrome by magnetic resonance imaging (MRI). The posterior reversible leukoencephalopathy syndrome subsided quickly after control of her hypertension and reinforcement of immunosuppressive treatment. In case 2, the patient also developed epileptic symptoms on the basis of GBM disease, and was given treatment similar to that of Case 1, so that the epileptic symptoms were controlled.

**Result:**

Reversible posterior leukoencephalopathy syndrome, especially when accompanied by cerebral hemorrhage, may lead to irreversible and lethal neurological abnormalities, and nephrologists should, therefore, be aware of the potential risk of reversible posterior leukoencephalopathy syndrome in patients with anti‐GBM disease. We can discuss the current two cases in the light of the previous literature.

## 1 INTRODUCTION

Anti‐glomerular basement membrane (GBM) disease, also known as Goodpasture's disease or syndrome and rapidly progressive glomerulonephritis type 1, is a rare, life‐threatening, small vessel vasculitis mediated by the abnormal production of anti‐GBM antibody elicited by autoimmune or alloimmune mechanisms, predominantly targeting the noncollagenous domain of the alpha 3 chain of type IV collagen in GBM, alveolar basement membrane, or both.^1^, ^2^ Anti‐GBM disease classically leads to renal dysfunction and pulmonary disease, characterized by rapidly progressive glomerulonephritis with or without pulmonary hemorrhage, with microscopic hematuria with proteinuria and increased urea nitrogen and serum creatinine in laboratory examination.^3^, ^4^ The progress of diagnostic serological testing, as well as clinical understanding of its pathogenesis and effective treatment strategies, mean that anti‐GBM disease can usually be controlled through immunosuppressive treatment, while its early diagnosis and effective immunosuppression can reduce the number of patients with end‐stage renal disease.^5^

Posterior reversible encephalopathy syndrome (PRES) is an acute neurological syndrome of heterogeneous etiologies grouped together based on similar findings on neuroimaging studies.^6^, ^7^ Presentation of PRES is characterized by generalized tonic‐clonic seizures, altered mental status, moderate‐to‐severe headaches, and visual disturbances, such as visual hallucinations and cortical blindness.^8^ It is caused by a variety of abnormalities in the endothelial function that ultimately result in vasogenic edema in the circulation of the central nervous system.^9^ This is reflected by the neuroimaging findings, which most often show symmetric reversible T2 high‐signal intensities in the occipital and parietal lobes detected by magnetic resonance imaging (MRI).^10^ An important proportion of patients with PRES present with anti‐GBM disease, and its complications, as their sole risk factors.^11^, ^12^, ^13^

Here, we report two cases of anti‐GBM disease with rapidly progressive glomerulonephritis (RPGN) and alveolar hemorrhage, with PRES and subcortical cerebral hemorrhage occurred during the treatment of anti‐GBM disease. Recent research has proposed a new hypothesis regarding the immunological mechanism by which anti‐GBM disease can result in PRES.^12^ They speculated that anti‐GBM antibodies might directly attack the cerebral vascular basement membrane. Consistent with previous reports in the literature, we also speculate that endothelial dysfunction leading to the development of PRES is caused not only by known risk factors such as cytotoxic agents, blood transfusions, or renal failure, but also by immunological abnormalities due to anti‐GBM antibody disease.

### 1.1 Case 1

A 40‐year‐old woman was hospitalized for the treatment of nausea, fever, and anorexia. No significant past history of any disease was mentioned. Twenty days before admission, she caught cold, developed high‐grade fever, followed by chills, dizziness, headache, nausea, vomiting, which were aggravated in the afternoon and at night, and lasted for 1–2 h. After taking ibuprofen and pain relieving tablets, the body temperature could drop, accompanied by slight pharyngodynia. The patient had tawny urine at the initial stage of fever without paying attention to whether it was accompanied by foam urine, without edema of both lower limbs, frequency of urination, urgency and pain of urination, change of urine volume, rash, joint pain, and oral ulcer. She visited a nearby hospital and was admitted for further evaluation. Laboratory data showed a white blood cell count of 11.73 × 10^9^/L, hemoglobin level 110 g/L, serum C‐reactive protein (CRP) level 250.98 mg/L, serum uric acid (UA) level 476.16 μmol/L, serum urea level 27.96 mmol/L, and serum creatinine level 413.7 μmol/L. Urine dip test revealed 1+ proteinuria and 3+ hematuria. The patient was initially treated with azithromycin, penicillin, and levofloxacin. However, her high‐grade fever persisted and serum levels of inflammatory indicators remained high despite without significant improvement. Therefore, she was transferred to our hospital for further diagnosis and treatment.

On hospitalization, the patient was alert with a blood pressure of 137/76 mmHg, respiratory rate 16/min, heart rate 78 teats/min, and body temperature 37.7°C. There were no positive signs and definite neurological findings at the time of admission. Laboratory study showed bandemia and mild microcytic hypochromic anemia. Blood hemoglobin level 75 g/L, white blood cell count 8.79 × 10^9^/L, serum CRP level 218.95 mg/L, serum UA level 373.00 μmol/L, serum urea level 18.02 mmol/L, and serum creatinine level 473.4 μmol/L. Urine dip test showed 1+ proteinuria and 3+ hematuria. The rate of deformed red blood cells was 60%, and the shape of deformed red blood cells was target type, small shadow red, occasionally granular casts. Serum immunological tests revealed normal serum levels of complements and immunoglobulins. Serum double‐stranded DNA, antinuclear antibody, myeloperoxidase antinuclear cytoplasmic antibody (ANCA), and proteinase 3‐ANCA were all negative. However, her anti‐GBM antibody titer was higher than the measurable upper limit of the test, showing (3+). To rule out bacterial infection, repeated blood culture tests were conducted, but no bacteria were detected in any of the blood‐culture bottles. Summary of blood and urine tests is shown in Table 1.

**Table 1:** Laboratory data on admission.

Laboratory findings	Values	Reference range	Units of measurement
*Complete blood count*			
Hemoglobin	75	110–150	g/L
Platelets	462	100–300	×10⁹/L
White blood cells	8.79	4–10	×10^9^/L
*Serum biochemistry*			
Albumin	25.33	38–60	g/L
Globulin	32.93	18–40	g/L
Alanine aminotransferase	9	0–40	U/L
Aspartate aminotransferase	17	0–40	U/L
Inorganic phosphate	1.79	0.7–1.5	mmol/L
Blood urea nitrogen	18.02	2.3–7	mmol/L
Creatinine	473.4	53–106	μmol/L
Cystatin C	4.41	0.51–1.09	mg/L
β2‐microglobulin	10.83	0–8	mg/L
Uric acid	373.00	150–410	mol/L
Glomerular filtration rate	9.01	>60.0	mL/min/1.732 m^2^
C‐reaction protein	218.95	0–8	mg/L
Erythrocyte sedimentation rate	96	0–15	mm/h
Procalcitonin	0.716	0–0.5	ng/mL
Brain natriuretic polypeptide	102.00	<100	pg/mL
*Serum immunological tests*			
Antiglomerular basement membrane antibody	3+	Negative	
Antineutrophil cytoplasmic antibody	Negative	Negative	
Antinuclear antibody	Negative	Negative	
Antidouble‐stranded DNA antibody	Negative	Negative	
*Coagulation*			
PT‐INR	1.39	0.8–1.1	
APTT	32.8	23–37	s
Fibrinogen	8.81	2.38–4.98	g/L
d‐dimer	1235	0–250	ng/mL

Abbreviations: APTT, activated partial thromboplastin time; PT‐INR, prothrombin time‐international normalized ratio.

Abdominal color Doppler ultrasound on admission showed fatty liver and bilateral kidney diffuse lesions. Additional chest computed tomography (CT) disclosed that a small amount of bilateral pleural effusion with adjacent lung tissue distension, indicating alveolar hemorrhage associated with anti‐GBM disease. Based on these findings, the patient was diagnosed with anti‐GMB disease.

Therefore, we chose to administer 500 mg intravenous methylprednisolone for 3 consecutive days, followed by daily treatment of 40 mg intravenous methylprednisolone. After that, the dosage was gradually reduced, and regular plasma exchange and hemofiltration treatment were started immediately. Cyclophosphamide 400 mg was administered intravenously on the 7th and 10th day after admission, respectively. Eight days after admission, ultrasound‐guided percutaneous renal biopsy was performed to assess glomerular abnormalities associated with the presence of anti‐GBM antibody. Light microscopic examination on a total of 13 glomeruli revealed some pathological abnormalities, including 2 focal segmental sclerosis, 2 macrocellular crescents, 6 cellular fibrous crescents, and 1 fibrous crescent. It was further found that the renal tubulointerstitium was moderately chronic with acute lesions, interstitial fibrosis 2+, focal tubule atrophy, focal tubule epithelial brush edge fell off and became flat, epithelial cells became vacuolar degeneration, and monocyte infiltration. There was no definite lesion in the arterioles. Immunofluorescence showed that immunoglobulin G (IgG) and fibrin were positive and diffusely distributed, which linearly deposited in vascular loops. However, there were no immune deposits for other immunoglobulins and complements. Furthermore, it was found by immunohistochemistry that Kappa, Lambda, C4d, and IgG were positive in vascular loops. Further, Congo red staining was performed and was negative.

On the 12th day after admission, the patient suddenly lost consciousness, failed to respond to the call, convulsed the whole body, frothed at the mouth, and hoisted his eyes. At this time, the patient was alert with a blood pressure of 210/132 mmHg, heart rate 154 beats/min, respiratory 32/min, blood oxygen saturation 80%, and developed dilated pupils of 5.0 mm on both sides, which were sensitive to light. An oropharyngeal airway was administered immediately, sputum was aspirated, and 10 mg of diazepam injection was given intravenously, nicardipine and sodium valproate were pumped by a micropump for 24 h, and 20 mg of diazepam infusion was given intravenously. Since then, the patient has intermittent unconsciousness, accompanied by intermittent convulsions, gibberish, dysphasia, so she continues to be treated with regular plasma exchange and hemofiltration, and sodium valproate, diazepam, phenobarbiterate, dexmedetomidine, and other antiepileptic treatment. Considering bidirectional affective disorder according to the patient's restlessness, risperidone, alprazolam and olanzapine were given for treatment. Urapidil and nifedipine sustained‐release tablets were taken for blood pressure. One day after initiating the antiepileptic drug, her consciousness became almost normal and her blindness resolved 2 days later. Reexamination of brain CT showed abnormal signals in bilateral fronto‐parietal occipital lobe, and the lesion area was significantly enlarged compared with that on admission, suggesting the possibility of reversible leukoencephalopathy in the posterior part of the brain. By long‐term electroencephalogram (EEG), typical sharp slow compound waves, about 3 Hz, 50–120 UV, were observed in the frontal region, without clinical seizure of epilepsy.

The patient was finally discharged with virtually no neurological sequelae on the 50th hospital day. Eight months after discharge, she continued to receive maintenance hemodialysis with no relapse of her anti‐GBM disease or seizures, under 10 mg/day of oral prednisolone treatment.

### 1.2 Case 2

A 77‐year‐old woman was presented with macroscopic hematuria for 2 months and fatigue for 1 month before admission. She had previously been diagnosed and treated for hypertension and palpitation in the past. She has cold and chills that had been admitted to nearby hospital as pneumonia, but here progressive nausea and vomiting caused her to transfer to Shanxi Bethune Hospital. Laboratory data showed a white blood cell count of 7.9 × 10^9^/L, hemoglobin level 96 g/L, serum urea level 24.6 mmol/L, and serum creatinine level 716.6 μmol/L. Urine dip test revealed proteinuria (1+) and hematuria (3+). Urinary sedimentation test showed numerous red blood cells per high power field (HPF), white blood cells 15–20/HPF. Her serum anti‐GBM antibody titer tested on admission was demonstrated to be very high (232.37 RU/mL), and therefore, the diagnosis of anti‐GBM disease was suggested. After 15 times of plasma exchange, the patient was reexamined for urea nitrogen (13.3 mmol/L), creatinine (369 μmol/L), and anti‐GBM antibody (89.84 RU/mL), and transferred to our hospital for further treatment.

On hospitalization, she was alert with a blood pressure of 165/82 mmHg, heart rate 74 beats/min, respiratory rate 20/min, and body temperature 36.2°C. On physical examination, her palpebral conjunctiva was anemic and pretibial pitting edema was present. There were no definite neurological findings on admission. Laboratory study revealed mild hypochromic microcytic anemia. Blood hemoglobin level 82 g/L, white blood cell count 3.28 × 109/L, serum CRP level 1.64 mg/L, serum UA level 321.20 μmol/L, serum urea level 18.75 mmol/L, and serum creatinine level 452.5 μmol/L. Urine dip test showed 2+ proteinuria and 2+ hematuria. The rate of deformed red blood cells was 55%, and the shape of deformed red blood cells was target type and small shadow red. Serum immunological tests revealed normal serum levels of complements and immunoglobulins. Serum double‐stranded DNA, myeloperoxidase ANCA, proteinase 3‐ANCA, and antinuclear antibody were all negative. However, her anti‐GBM antibody titer was above the upper measurable limit of the test (1+). Summary of blood and urine tests is shown in Table 2.

**Table 2:** Laboratory data on admission.

Laboratory findings	Values	Reference range	Units of measurement
*Complete blood count*			
Hemoglobin	82	110–150	g/L
Platelets	36	100–300	×10^9^/L
White blood cells	3.28	4–10	×10^9^/L
*Serum biochemistry*			
Albumin	30.78	38–60	g/L
Globulin	21.07	18–40	g/L
Alanine aminotransferase	14.53	0–40	U/L
Aspartate aminotransferase	16.30	0–40	U/L
Blood urea nitrogen	18.75	2.3–7	mmol/L
Creatinine	452.5	53–106	μmol/L
Cystatin C	10.49	0.51–1.09	mg/L
β2‐microglobulin	23.24	0–8	mg/L
Uric acid	321.20	150–410	mol/L
Glomerular filtration rate	8.47	>60.0	mL/min/1.732 m^2^
C‐reaction protein	1.64	0–8	mg/L
Erythrocyte sedimentation rate	8	0–15	mm/h
Brain natriuretic polypeptide	369.00	<100	pg/ml
*Serum immunological tests*			
Antiglomerular basement membrane antibody	1+	Negative	
Antineutrophil cytoplasmic antibody	Negative	Negative	
Antinuclear antibody	Negative	Negative	
Antidouble‐stranded DNA antibody	Negative	Negative	
*Coagulation*			
PT‐INR	1.39	0.8–1.1	
APTT	32.8	23–37	s
Fibrinogen	1.41	2.38–4.98	g/L
d‐dimer	867	0–250	ng/mL

Abbreviations: APTT, activated partial thromboplastin time; PT‐INR, prothrombin time‐international normalized ratio.

Abdominal color Doppler ultrasound on admission showed cholecystitis with cholesterol crystals in the gallbladder wall and bilateral kidney diffuse lesions. Additional chest CT disclosed that bulla in the lower lobe of the right lung and left pneumonia are possible.

After admission, she received oral prednisolone (40 mg daily), arotinolol (10 mg twice daily), sacubitril valsartan (50 mg daily), mycophenolate mofetil (0.5 g twice daily), and anti‐infection and symptomatic supportive treatment from the second hospital day. She also underwent seven courses of plasma exchange. The next day, after falling, the patient suddenly had limb convulsions, hanging eyes, trismus, flexion of both upper limbs, and straight of both lower limbs. At this time, the blood pressure was 185/85 mmHg, the heart rate was 108 beats/min, the respiration was 24 beats/min, the blood oxygen saturation was 97%, the pupil size was normal (both sides were 3.0 mm), and the patient was sensitive to light. Turn his head to one side immediately, and insert a towel into his mouth to prevent a tongue bite. It relieved spontaneously after 3 min, and a one‐time oropharyngeal airway was immediately changed to assist ventilation. One hour later, the patient convulsed again. At this time, the blood pressure was 189/84 mmHg, the heart rate was 98 beats/min, the respiration was 20 beats/min, the blood oxygen saturation was 98%, the pupil size was normal (both sides were 3.0 mm), and the patient was sensitive to light. Restrain the limbs immediately, inject diazepam intramuscularly, and pump sodium valproate into the body with a micro pump at 2 mL/h. It relieved spontaneously after 2 min. After that, there were four intermittent attacks, and the maximum blood pressure reached 182/105 mmHg. The convulsion was relieved after increasing the pump speed of sodium valproate, the static push of diazepam, and a micropump of 3 mL/h of nicoldipine.

The patient was finally discharged with virtually no neurological sequelae on the 80th hospital day. A total of 17 hemofiltration sessions were administered during hospitalization, and screening for anti‐GBM was negative at discharge. Seventeen months after discharge, she continued to receive maintenance hemodialysis with no relapse of her anti‐GBM disease or seizures, under 10 mg/day of oral prednisolone therapy.

## 2 DISCUSSION

RPGN is clinically manifestations as a rapidly progressive renal failure and pathologically as crescentic and necrotizing lesions with infiltration of inflammatory cells in the glomeruli.^14^ The autoimmune development of antibodies towards type IV collagen in the glomerular and alveolar basement membranes leads to patients typically presenting with RPGN and pulmonary hemorrhage.^15^, ^16^, ^17^ Evidence of anti‐GBM antibodies in serum or histologically is required for diagnosis.^18^ Treatment mainly involves the use of apheresis and intensive, strong immunosuppression such as cyclophosphamide.^19^

PRES is a neurological condition characterized by headache, seizure, impaired visual acuity or blindness, focal weakness, and occasionally focal neurological deficits corresponding to the affected lesions of the brain.^20^ It is caused by a variety of abnormalities in the endothelial function that ultimately result in vasogenic edema in the circulation of the central nervous system. This is reflected by the neuroimaging findings, which most often show reversible parieto‐occipital edema. An important proportion of patients with PRES present with nephritis, and its complications, as their sole risk factors. Notably, the neurological abnormalities caused by PRES are occasionally irreversible and lethal, especially in patients who develop brain hemorrhage and status epilepticus

It is known that there are several case reports of PRES occurring during the course of anti‐GBM disease.^11^, ^21^ The frequency of such reports in anti‐GBM disease is higher compared to other diseases that also lead to acute renal failure.^20^ Particularly, Taniguchi and Hanaoka have proposed a new hypothesis regarding the immunological mechanism by which anti‐GBM disease can result in PRES.^12^ They speculated that anti‐GBM antibodies might directly attack the cerebral vascular basement membrane. Anti‐GBM antibodies are known to recognize α3[IV]NC1, mainly present in the glomerular and alveolar basement membranes. While the cerebral vascular basement membrane is composed of α2[IV]NC1, not α3[IV]NC1, it initially appears that anti‐GBM antibodies should not damage it. However, Zhao et al. have reported partial cross‐reactivity of anti‐GBM antibodies with α1[IV]NC1, α2[IV]NC1, and α5[IV]NC1.^13^ Moreover, the presence of α3[IV]NC1 in the choroid plexus has been reported, making it molecularly plausible that the central nervous system could be a target for anti‐GBM antibodies.

Epilepsy is a common and frequently‐occurring disease caused by the highly synchronous paradoxical discharge of cerebral cortex neurons, which clinical manifestations are characterized by seizure, transient, repetitive, and stereotypic.^22^ Different locations of paradoxical discharge neurons and different ranges of paradoxical discharge lead to different seizure forms in patients, which can be manifested as sensory, motor, consciousness, mental, behavioral, autonomic nerve dysfunction, or both.^23^ In general tonic‐clonic seizure, the main clinical features are loss of consciousness and clonic appearance after bilateral tonicity. Both of the two patients in our case report had clinical manifestations of seizures at the time of seizure, which were confirmed by their EEG results.^24^

The epilepsy in Case 1 was considered to be induced by the PRES, which clinically shows epilepsy, and vasogenic edema of the parieto‐occipital lobe, caused mainly by included hypertension, eclampsia/pre‐eclampsia, and various kidney diseases.

It can be an acute or subacute onset, and its main clinical manifestations are headache, epileptic seizure, visual disorder, consciousness disorder, mental disorder, and so forth.^13^ This disease is radiographic changes are mainly in the posterior white matter of the brain, most of which can be recovered in a short time. MRI usually shows long T1 and long T2 signals in the posterior part of the brain (mainly in the parietal and occipital lobes) on one or both sides, which is a high signal on T2FLAIR, and can also occur in any part of the brain. Diffusion‐weighted imaging (DWI) is mostly isointense, and the apparent diffusion coefficient value is generally increased.^20^ Up to now, there is no definite pathogenesis that can be explained clearly, and it mainly tends to be explained by the high perfusion theory and the low perfusion theory.

In Case 1, the blood pressure of the patient was 210/132 mmHg at the time of the attack, who also was a patient with anti‐GBM type rapidly progressive nephritis. Before the seizure, the patient's serum creatinine reached 536.6 μmol/L, with acute onset, blurred vision, epilepsy, confusion, restlessness, gibberish and other manifestations. Cranial MRI showed abnormal long T1 and T2 signals in the left frontal and bilateral temporooccipital lobes, with a normal magnetic resonance arterial angiography, which was consistent with the diagnosis of reversible posterior leukoencephalopathy syndrome. After active control of blood pressure, regular dialysis, and antiepileptic treatment, the patient's consciousness turned clear, and no seizure occurred for several consecutive days.

In Case 2, the epileptic patient was diagnosed with uremic encephalopathy after discharge. Combined with the patient's MRI, it was suggested that scattered speckled mild long T2 high signal shadows could be seen in the white matter of bilateral cerebral hemispheres, paraventricular areas, and right basal ganglia, with vague outlines, but no abnormal high signal shadows were found in DWI. Since the patient had a history of hypertension and the highest blood pressure at the time of attack was 195/103 mmHg, it was considered as reversible posterior leukoencephalopathy syndrome. It may be that the lesion site of Case 2 was not located in the classic part of the parietal occipital lobe, which led to the radiologist ignoring the possibility of this diagnosis.

Currently, the recognized pathogenesis of PRES mainly includes the following two aspects, including cerebrovascular automatic regulation mechanism disorders and endothelial cell dysfunction caused by various reasons.^25^, ^26^ When the cerebrovascular autoregulation mechanism is blocked, excessive blood pressure breaks through the automatic regulation range of cerebral blood flow (usually 50–150 mmHg), resulting in brain hyperperfusion.^27^ The posterior cerebral circulation area is more vulnerable to damage due to weak sympathetic regulation. However, 15%–20% of PRES patients have normal or low blood pressure. Of course, it is also possible that high blood pressure values are not monitored or that the upper limit of automatic regulation of cerebral blood flow varies from person to person. When endothelial cell dysfunction occurs, a large number of inflammatory cytokines are released and the expression of adhesion molecules increases, resulting in endothelial cell swelling and increased vascular permeability, and finally brain edema.^28^ Therefore, we speculate that the cause of PRES in anti‐GBM disease may be vasogenic edema secondary to endothelial cell dysfunction.

The treatment of PRES mainly focuses on the etiology, including hypotension, early withdrawal of cytotoxic drugs, treatment of the primary disease, antiepilepsy, dehydration and cranial pressure reduction, and so forth.^9^ In cases of PRES related to hypertension, rapid drop in blood pressure (no more than 25% drop within the first few hours) and large fluctuations in blood pressure should be avoided during antihypertensive treatment, and continuous intravenous pumping of antihypertensive drugs is preferred.^9^, ^29^ In cases related to renal disease, renal function should be closely monitored and electrolyte imbalances corrected.^29^ Most patients (75%–90%) can recover completely, with complete improvement of clinical symptoms, and some patients may take longer, but 3%–19% of patients die.^9^, ^28^, ^29^

In summary, we report two patients with anti‐GBM disease with reversible posterior leukoencephalopathy syndrome, accompanied by epileptic during the therapeutic course. Cranial MRI indicated vasogenic edema in parieto‐occipital lobe. After treatment with antihypertensive drugs, the patient did not have any neurological symptoms again, and the radiographic lesions completely disappeared after reexamination. This is a classic case of successful treatment of anti‐GBM disease combined with PRES, which is likely caused by vasogenic edema secondary to endothelial cell dysfunction. Although the patient has risk factors for death such as renal insufficiency, the prognosis of the patient is good due to early diagnosis, and timely antihypertensive and antiepileptic treatment. This case highlights the importance of prompt recognition and treatment of PRES, particularly in patients with anti‐GBM disease, and underscores the need for further research to elucidate the underlying mechanisms and optimal management strategies for this condition.

Further case series and observational studies may be needed to elucidate the mechanism underlying the possible coexistence of anti‐GBM disease and reversible posterior leukoencephalopathy syndrome.

## AUTHOR CONTRIBUTIONS

**Chongyang Han**: Resources. **Xiangrong Cui**: Software; visualization. **Zhicheng Tan**: Funding acquisition. **Yafeng Li**: Writing—original draft.

## CONFLICT OF INTEREST STATEMENT

The authors declare no conflict of interest.

## ETHICS STATEMENT

The present work is original and has not been published elsewhere in any form or language. The patient provided written consent for the publication of her clinical information.

## Supporting information

Supporting information.
