# Supplementary material for: Antiglomerular basement membrane antibody type rapidly progressive glomerulonephritis with seizures: Two cases and literature review
Source: Immun Inflamm Dis. 2023 Nov 8;11(11):e1074. doi: 10.1002/iid3.1074 (PMC10632087; doi:10.1002/iid3.1074)
Supplement: Supplementary file 1 — Supporting information. [file IID3-11-e1074-s001.docx]

**Author accounts and email addresses**

**1. Chongyang Han:**

Department of Nephrology, Shanxi Provincial People's Hospital (Fifth Hospital) of Shanxi Medical University; Taiyuan, China

**Email:** start207@163.com

**ORCID:** 0009-0005-9319-1487; <https://orcid.org/0009-0005-9319-1487>

**Published Articles**

(1) Jing X, Cui X, Liang H, Hao C, Yang Z, Li X, Yang X, Han C. CD24 is a Potential Biomarker for Prognosis in Human Breast Carcinoma. Cell Physiol Biochem. 2018;48(1):111-119. doi: 10.1159/000491667. Epub 2018 Jul 12. PMID: 30001552.

(2) Jing X, Liang H, Cui X, Han C, Hao C, Huo K. Long noncoding RNA CCAT2 can predict metastasis and a poor prognosis: A meta-analysis. Clin Chim Acta. 2017 May;468:159-165. doi: 10.1016/j.cca.2017.03.003. Epub 2017 Mar 3. PMID: 28263738.

(3) Jing X, Cui X, Liang H, Hao C, Han C. Diagnostic accuracy of ELISA for detecting serum Midkine in cancer patients. PLoS One. 2017 Jul 7;12(7):e0180511. doi: 10.1371/journal.pone.0180511. PMID: 28686647; PMCID: PMC5501560.

**2. Xiangrong Cui**

Reproductive Medicine Center, The affiliated Children's Hospital of Shanxi Medical University, Children's Hospital of Shanxi, Shanxi Maternal and Child Health Hospital, Taiyuan, 030001, China

**Email:** cuixiangrong094030@163.com

**ORCID:** 0000-0001-6328-6526; <https://orcid.org/0000-0001-6328-6526>

**Published Articles**

(1) Jing X, Cui X, Liang H, Hao C, Yang Z, Li X, Yang X, Han C. CD24 is a Potential Biomarker for Prognosis in Human Breast Carcinoma. Cell Physiol Biochem. 2018;48(1):111-119. doi: 10.1159/000491667. Epub 2018 Jul 12. PMID: 30001552.

(2) Jing X, Liang H, Cui X, Han C, Hao C, Huo K. Long noncoding RNA CCAT2 can predict metastasis and a poor prognosis: A meta-analysis. Clin Chim Acta. 2017 May;468:159-165. doi: 10.1016/j.cca.2017.03.003. Epub 2017 Mar 3. PMID: 28263738.

(3) Jing X, Cui X, Liang H, Hao C, Han C. Diagnostic accuracy of ELISA for detecting serum Midkine in cancer patients. PLoS One. 2017 Jul 7;12(7):e0180511. doi: 10.1371/journal.pone.0180511. PMID: 28686647; PMCID: PMC5501560.

(4) Cui X, Wang H, Wu X, Huo K, Jing X. Increased expression of KPNA2 predicts unfavorable prognosis in ovarian cancer patients, possibly by targeting KIF4A signaling. J Ovarian Res. 2021 May 25;14(1):71. doi: 10.1186/s13048-021-00818-9. PMID: 34034774; PMCID: PMC8152344.

(5) Cui X, Su H, Yang J, Wu X, Huo K, Jing X, Zhang S. Up-regulation of MTHFD2 is associated with clinicopathological characteristics and poor survival in ovarian cancer, possibly by regulating MOB1A signaling. J Ovarian Res. 2022 Feb 8;15(1):23. doi: 10.1186/s13048-022-00954-w. PMID: 35135596; PMCID: PMC8827288.

(6) Cui X, Wu X, Wang H, Zhang S, Wang W, Jing X. Genetic of preimplantation diagnosis of dysmorphic facial features and intellectual developmental disorder (CHDFIDD) without congenital heart defects. Mol Genet Genomic Med. 2022 Feb;10(2):e1863. doi: 10.1002/mgg3.1863. Epub 2022 Jan 16. PMID: 35034425; PMCID: PMC8830809.

(7) Jing X, Liang H, Hao C, Yang X, Cui X. Overexpression of MUC1 predicts poor prognosis in patients with breast cancer. Oncol Rep. 2019 Feb;41(2):801-810. doi: 10.3892/or.2018.6887. Epub 2018 Nov 27. PMID: 30483806; PMCID: PMC6313072.

**3. Zhicheng Tan**

Department of Nephrology, Shanxi Provincial People's Hospital (Fifth Hospital) of Shanxi Medical University; Taiyuan, China

**Email:** taiyuantzc@163.com

**Published Articles**

(1) Tan Z, Shi Y, Yan Y, Liu W, Li G, Li R. Impact of endogenous hydrogen sulfide on toll-like receptor pathway in renal ischemia/reperfusion injury in rats. Ren Fail. 2015 May;37(4):727-33. doi: 10.3109/0886022X.2015.1012983. Epub 2015 Feb 20. PMID: 25697231.

**4. Yafeng Li**

Department of Nephrology, Shanxi Provincial People's Hospital (Fifth Hospital) of Shanxi Medical University; Taiyuan, China

Shanxi Provincial Key Laboratory of Kidney Disease; Taiyuan, China

**Email:** dr.yafengli@gmail.com

**Published Articles**

(1) Song W, Zhang S, Li X, Gao C, Cai J, Li Y. Editorial: Relationship between intestinal microbiome and vasculitis. Front Cell Infect Microbiol. 2023 Apr 4;13:1165730. doi: 10.3389/fcimb.2023.1165730. PMID: 37082712; PMCID: PMC10111023.

(2) Song W, Qin Z, Hu X, Han H, Li A, Zhou X, Li Y, Li R. Using Bayesian networks with Tabu-search algorithm to explore risk factors for hyperhomocysteinemia. Sci Rep. 2023 Jan 28;13(1):1610. doi: 10.1038/s41598-023-28123-z. PMID: 36709366; PMCID: PMC9884210.

(3) Song W, Qiu L, Qing J, Zhi W, Zha Z, Hu X, Qin Z, Gong H, Li Y. Using Bayesian network model with MMHC algorithm to detect risk factors for stroke. Math Biosci Eng. 2022 Sep 19;19(12):13660-13674. doi: 10.3934/mbe.2022637. PMID: 36654062.

(4) Hu X, Fan R, Song W, Qing J, Yan X, Li Y, Duan Q, Li Y. Landscape of intestinal microbiota in patients with IgA nephropathy, IgA vasculitis and Kawasaki disease. Front Cell Infect Microbiol. 2022 Dec 16;12:1061629. doi: 10.3389/fcimb.2022.1061629. PMID: 36590596; PMCID: PMC9800820.

(5) Cheng T, Zhi H, Liu Y, Zhang S, Song Z, Li Y. Dual Anti-Glomerular Basement Membrane and Anti-Neutrophil Cytoplasmic Antibodies-Positive Rapidly Progressive Glomerulonephritis with Rheumatoid Arthritis and Sjogren's Syndrome: A Case Report and Literature Review. J Clin Med. 2022 Nov 16;11(22):6793. doi: 10.3390/jcm11226793. PMID: 36431270; PMCID: PMC9697768.

**5. Yufeng Qiao：**

Department of Nephrology, Shanxi Provincial People's Hospital (Fifth Hospital) of Shanxi Medical University; Taiyuan, China

**Email:** [qiaoyufeng416@163.com](mailto:qiaoyufeng416@163.com)

**ORCID:** 0009-0004-7788-9204; <https://orcid.org/0009-0004-7788-9204>

**Published Articles**

(1) Zhou X, Xue F, Wang H, Qiao Y, Liu G, Huang L, Li D, Wang S, Wang Q, Li L, Li R. The quality of life and associated factors in patients on maintenance hemodialysis - a multicenter study in Shanxi province. Ren Fail. 2017 Nov;39(1):707-711. doi: 10.1080/0886022X.2017.1398095. PMID: 29141472; PMCID: PMC6446155.

(2) Qiao YF, Guo WJ, Li L, Shao S, Qiao X, Shao JJ, Zhang Q, Li RS, Wang LH. Melatonin attenuates hypertension-induced renal injury partially through inhibiting oxidative stress in rats. Mol Med Rep. 2016 Jan;13(1):21-6. doi: 10.3892/mmr.2015.4495. Epub 2015 Nov 2. PMID: 26531807; PMCID: PMC4686099.

(3) Qiao YF, Pang DZ, Lu JF, Hu BY, Liu D, Zhou GF, Yang B, Li RS, Jiang YS. [Effect of potassium iodide on prevention of experimental lead nephropathy and expression of nuclear factor-kappaB and fibronectin]. Zhonghua Lao Dong Wei Sheng Zhi Ye Bing Za Zhi. 2009 Dec;27(12):747-52. Chinese. PMID: 21141136.
